# The pathology of lumbosacral lipomas: macroscopic and microscopic disparity have implications for embryogenesis and mode of clinical deterioration

**DOI:** 10.1111/his.13469

**Published:** 2018-03-15

**Authors:** Victoria Jones, Victoria Wykes, Nicki Cohen, Dominic Thompson, Tom S Jacques

**Affiliations:** ^1^ Developmental Biology and Cancer Programme UCL Institute of Child Health London UK; ^2^ Department of Neurosurgery Great Ormond Street Hospital NHS Trust London UK; ^3^ Department of Histopathology Great Ormond Street Hospital NHS Trust London UK; ^4^ Department of Histopathology Kings College London London UK

**Keywords:** adipocytes, conus medullaris, dysraphism, lipomyelomeningocele, lumbosacral lipoma, spinal cord untethering, tethered cord syndrome

## Abstract

**Aims:**

Lumbosacral lipomas (LSL) are congenital disorders of the terminal spinal cord region that have the potential to cause significant spinal cord dysfunction in children. They are of unknown embryogenesis with variable clinical presentation and natural history. It is unclear whether the spinal cord dysfunction reflects a primary developmental dysplasia or whether it occurs secondarily to mechanical traction (spinal cord tethering) with growth. While different anatomical subtypes are recognised and classified according to radiological criteria, these subtypes correlate poorly with clinical prognosis. We have undertaken an analysis of surgical specimens in order to describe the spectrum of histological changes that occur and have correlated the histology with the anatomical type of LSL to determine if there are distinct histological subtypes.

**Methods and results:**

The histopathology was reviewed of 64 patients who had undergone surgical resection of LSL. The presence of additional tissues and cell types were recorded. LSLs were classified from pre‐operative magnetic resonance imaging (MRI) scans according to Chapman classification. Ninety‐five per cent of the specimens consisted predominantly of mature adipocytes with all containing thickened bands of connective tissue and peripheral nerve fibres, 91% of samples contained ectatic blood vessels with thickened walls, while 22% contained central nervous system (CNS) glial tissue. Additional tissue was identified of both mesodermal and neuroectodermal origin.

**Conclusions:**

Our analysis highlights the heterogeneity of tissue types within all samples, not reflected in the nomenclature. The diversity of tissue types, consistent across all subtypes, challenges currently held notions regarding the embryogenesis of LSLs and the assumption that clinical deterioration is due simply to tethering.

## Introduction

Lumbosacral lipomas (LSL) of the conus medullaris are a common form of spinal malformation. Diagnosis is made typically in infants on the basis of a midline lumbosacral swelling, sometimes accompanied by local cutaneous manifestations including dermal sinus, skin appendage and capillary haemangioma.^1^ At the time of diagnosis there may already be features of distal spinal cord dysfunction, including distal lower limb weakness and asymmetry, talipes deformities and features of neurogenic sphincter impairment; however, as many as 40% of cases are ostensibly asymptomatic at birth. Ultimately, over time all patients are at risk of new or progressive neurological deterioration.^2^, ^3^, ^4^ The role of resection of the lipoma and untethering of the spinal cord in averting neurological and urological deterioration is controversial.^5^, ^6^ The essence of this controversy is whether the neurological and urological disability is a result of secondary injury to the terminal spinal cord and cauda equina through a process of mechanical tethering – and thus potentially amenable to surgery – or whether dysfunction is a result of a primary inherent malformation.

**Figure 1:**
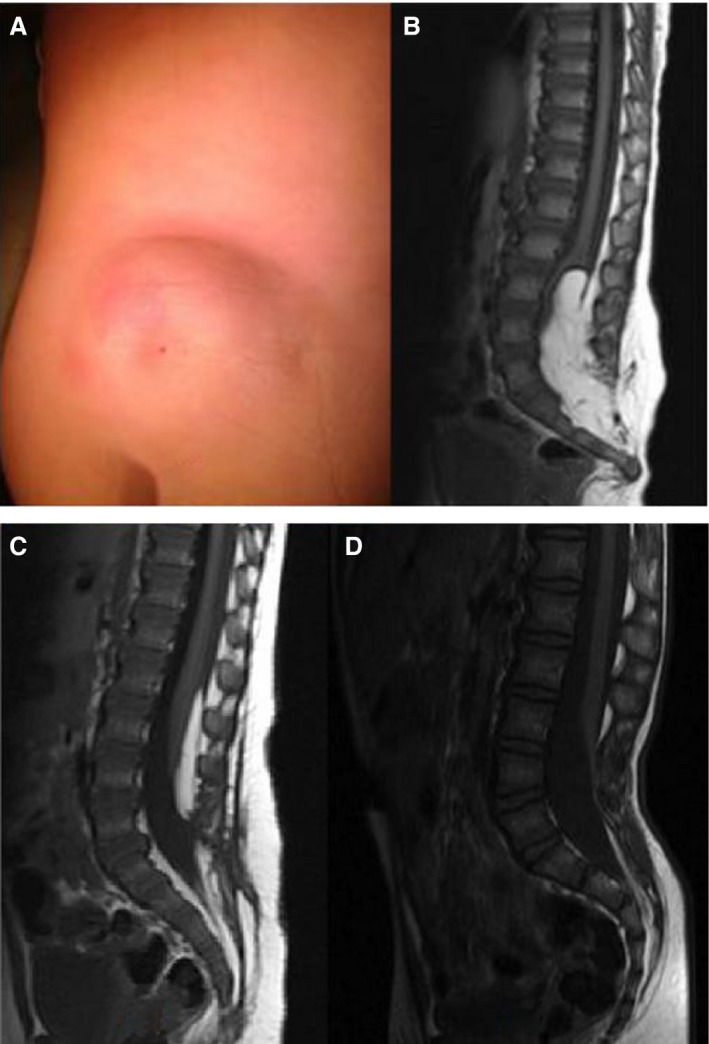
**A**, Lumbosacral lipoma with cutaneous dimple. **B–D**, Sagittal T1 magnetic resonance imaging (MRI) illustrating different anatomical subtypes of lumbosacral lipomas as described by Chapman. Lipoma tissue is closely adherent to splayed neural tissue forming a lipoma–neuronal placode; the lipoma extends through a defect in the posterior dura and vertebral column, before becoming continuous with the subcutaneous fat. Lumbosacral lipomas (LSLs) are classified based on their radiological appearance and relationship to the conus. **C**, Dorsal LSL: the interface between the lipoma and spinal cord is above the level of the conus. **B**, Transitional LSL: the interface includes the conus and the lipoma extends into elements of the cauda equina. **D**, Caudal LSL: the lipoma extends from the tip of the conus to the end of the thecal sac. Caudal LSL may be referred to alternatively as terminal or filar LSL. Pang *et al*. have recently describe a chaotic form that is most allied to the transitional type, but where the lipoma extends ventrally to the placode and nerve roots.^8^ [Colour figure can be viewed at http://wileyonlinelibrary.com]

Magnetic resonance imaging (MRI) is used to confirm the diagnosis and classify the LSL, depending on its relationship to the conus.^7^, ^8^ Although widely used, the classification of LSL is somewhat difficult to apply in practice and, according to most published series, the correlation between LSL type and neurological prognosis has been poor (Figure 1).

It has been established for almost 100 years that congenital spinal lipomas are different from lipomas at other sites.^4^ However, despite a wealth of publications during that time on the management and clinical presentation of spinal lipomas, there is surprisingly little in the literature about the histopathology, with one large series, two smaller series^9^, ^10^, ^11^, ^12^, ^13^ and a number of case reports often describing the bizarre and unusual.

LSLs are characterised by mature adipocytes, both microscopically and metabolically, surrounded by thickened bands of connective tissue and containing a diverse range of different cell types present from all three germ layers.^14^, ^15^, ^16^ This pathological heterogeneity within LSL has implications both for our understanding of their embryogenesis and the mechanisms underlying clinical deterioration. There have been no previous attempts to correlate histological findings to clinical or radiological features.

A large‐scale analysis of 671 patients over 22 years looked collectively at spinal lipomas of the filum and of the conus, and found that 77% were more complex lesions containing more than just adipocytes and collagen bands.^9^, ^11^, ^14^

Walsh *et al*. looked at 20 patients, and again a diverse group was considered including intradural lipomas. This paper showed principally ‘the presence of large, rather monotonous sheets of mature fat‐cells and thick strands of connective tissue. Numerous thin‐walled blood vessels were also seen’, but 25% (five cases) demonstrated a more diverse range of cell types.^10^ The two histopathology series above include lipomas within all radiological subsets.

We present here an intermediate‐size series of LSLs including detailed histopathological analysis of post‐operative samples. Unlike the above‐described series we have grouped our data based on radiological classification to test the hypothesis that there is a difference in histopathology between different anatomical subtypes.

## Materials and methods

Sixty‐four patients underwent resection of LSL and untethering of the spinal cord at Great Ormond Street Hospital between 1998 and 2010. Tissue was removed as part of planned surgical resection and placed in formalin. Routine histopathology sections were performed with haematoxylin and eosin (H&E) staining and immunostaining as part of standard diagnostic analysis. Analysis was performed within the histopathology department by a senior neuropathologist with experience in viewing LSLs (N.C.). Each specimen was analysed for the presence and frequency of tissue and cell type.

**Figure 2:**
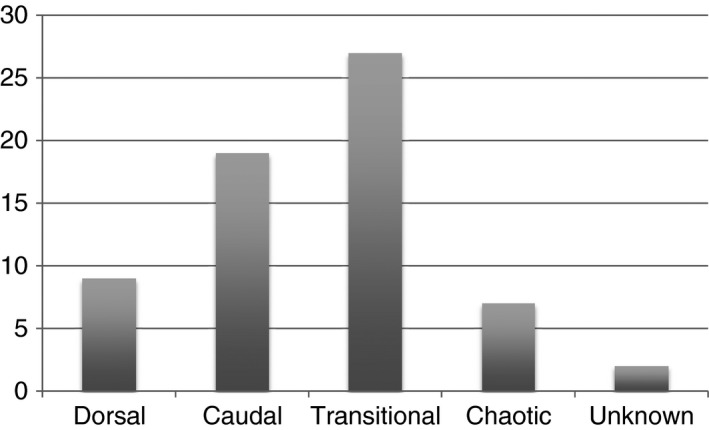
Breakdown of radiological subtypes. For description of classification see legend to Figure 1. It was not possible to classify two cases due to insufficient imaging.

Pre‐operative radiological MRI was reviewed in all cases. Only complex conus region lipomas were included and T1‐weighted axial and sagittal images were used to classify the LSLs as dorsal, caudal, transitional or chaotic. Teratomas and thickened (fatty) filums were excluded (Figure 2).

During the period of this study surgical technique comprised untethering of the spinal cord and subtotal resection of the LSL, leaving a cuff of lipoma adjacent to the neural placode. Neural elements would therefore not be anticipated in the surgical specimens.

Results are stated as percentage, and standard error was calculated to determine the 95% confidence interval (CI) (expressed in brackets).

The study was approved by Great Ormond Street Hospital and UCL Institute of Child Health Research and Development Office (16DD01).

## Results

Of the 64 specimens, 95.3% (95% CI = 87.98) consisted predominantly of mature adipocytes, while the other specimens had a greater proportion of immature adipocytes. All specimens had thickened bands of connective tissue within the adipose tissue. Within this connective tissue, all specimens had small peripheral nerve fibres present; 21.9% (95% CI ± 10.1) of specimens had central nervous system (CNS) glial cells present within the connective tissue.

**Figure 3:**
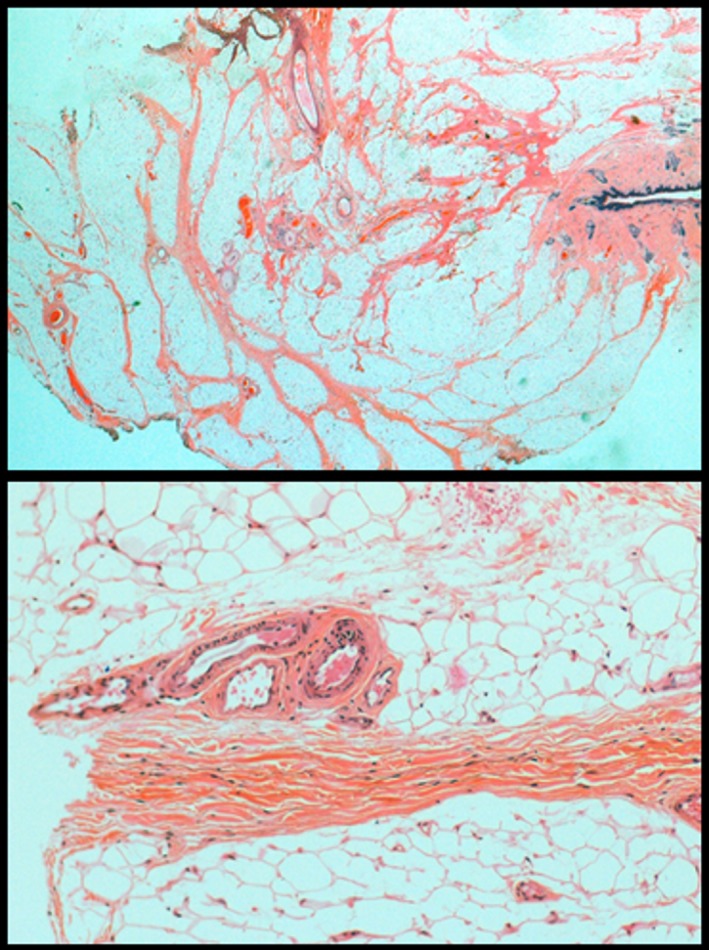
Histology slides of lipoma specimens demonstrating prominent blood vessels; 91% showed blood vessels focused upon the adipose with enlarged lumina and thickened walls and 74% showed similar vessels extending towards the base of the surgical resection adjacent to skeletal muscle.

Of the 64 specimens, 90.6% (95% CI ± 7.0) contained blood vessels with enlarged lumina and thickened walls. In 25.9% (95% CI ± 11.3) of these 58 specimens, these vessels were only located deep within the substance of the adipose tissue rather than at the presumed lipoma boundary. However, two of the cases that did not contain abnormal vessels showed deep vessels at the lipoma boundary (Figure 3). Of the 64 specimens, 48.4% (95% CI ± 12.2) demonstrated skeletal muscle surrounded by adipose tissue.

### Cellular diversity

A range of other tissue types were also identified with varying degrees of maturity; 15.6% (95% CI ± 8.9) contained glial CNS type tissue (as opposed to individual glial cells commented on above), 14.1% (95% CI ± 8.5) contained meningeal cells, 12.5% (95% CI ± 8.1) contained inflammatory cells (half of which were acute perivascular inflammation in keeping with prolonged surgery, while the other half demonstrated established inflammation with multinucleated/giant cells present), and 9.4% (95% CI = 4.4.19) contained ganglion cells.

In total, 22 (95% CI = 34 ± 11.6) specimens demonstrated differentiated structures, ranging from a cavity lined with meningeal tissue (seven), haemangiomas (two), cavity lined with ependymal tissue (three), lymph node (one), peripheral nerve bundles (six), bone marrow (two), bone (three) and Pacinian corpuscle (five).

The number of blocks analysed for each specimen ranged from one to nine. The diversity of cell and tissue types detected rose steadily and peaked at four blocks. Thereafter, analysis of further blocks did not to add any more findings, with an average number of additional cell types in a sample not rising above 4.1 (Supporting Information).

### Relationship to dermis

Of the 64 surgical specimens reviewed, 18 contained overlying epidermis and dermis. Epidermis was excised if there was a suspected sinus tract/pit or to aid skin closure; 50 ± 21% of these samples demonstrated a dermal pit. In 77.8 ± 19.2% adnexal structures were identified within the superficial adipose, regardless of whether it connected to the main adipose tissue of the lipoma. In 66.7 ± 21.8%, adipose was identified within the overlying dermis.

### Analysis by radiological classification

**Figure 4:**
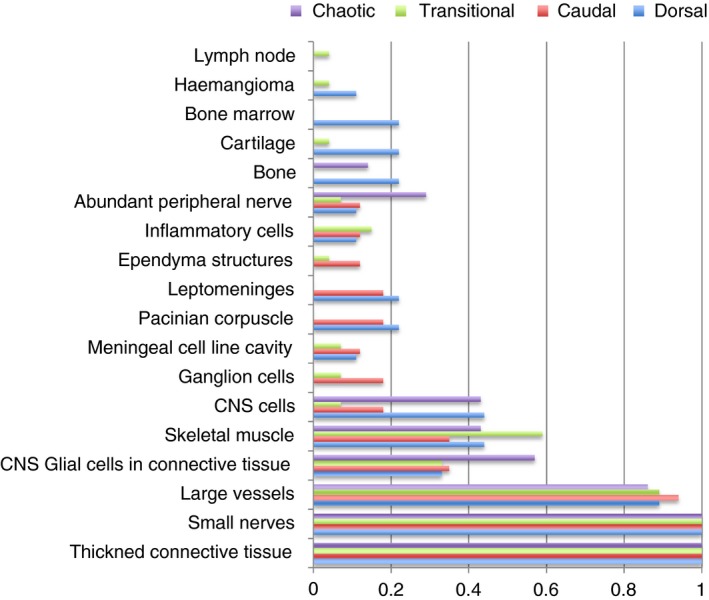
Results as per radiological subtype, expressed as a proportion. For statistical analysis see Supporting Information.

**Figure 5:**
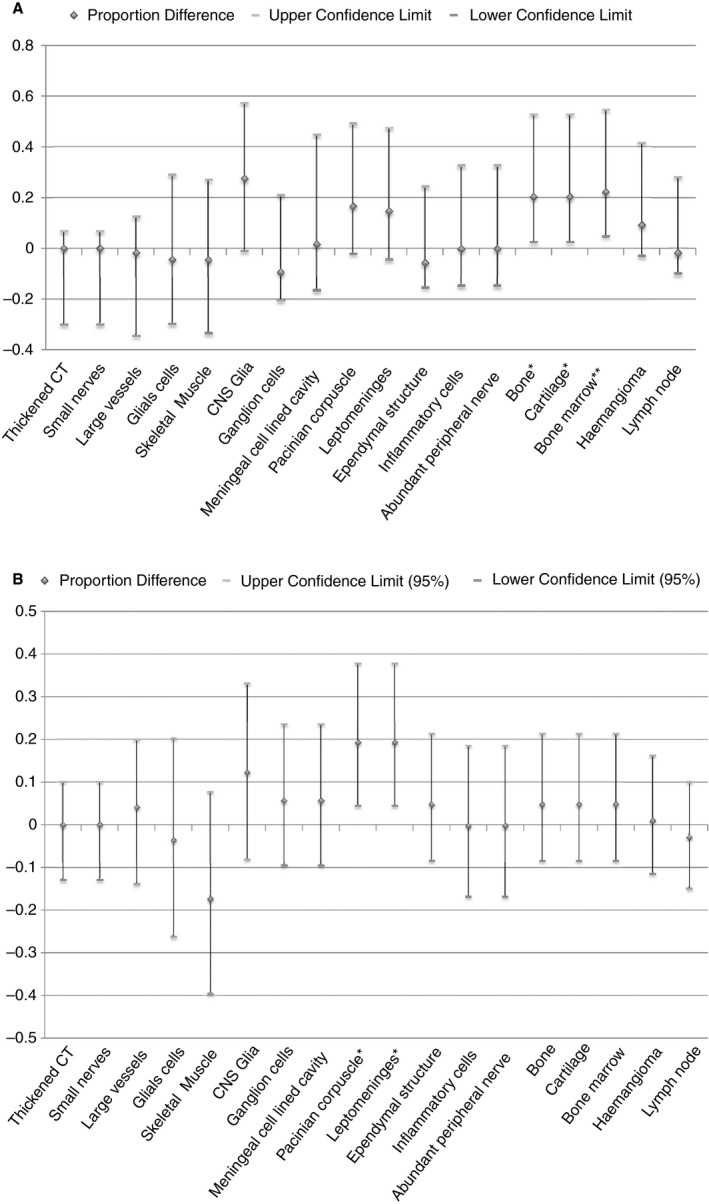
**A**, Subtypes of lipoma were grouped into those proposed to be due to a defect in primary neurulation (dorsal) and those proposed to be due to a defect in secondary neurulation (caudal, transitional and chaotic). Differences in proportion of different cell/tissue types detected were calculated along with 95% confidence intervals (CI) of the difference. *Values which show significant difference at the 95% CI. This significance is lost at 99% CI for the presence of bone and cartilage but not bone marrow; **difference = 0.222 (0.013, 0.635). **B**, Subtypes of lipoma were grouped into ‘simple’ (dorsal and caudal) and ‘complex’ (transitional and chaotic). Difference in proportion of different cell/tissue types detected was calculated along with 95% CI of the difference. *Values which show significant difference at the 95% CI. This significance is lost at 99% CI for the presence of Pacinian corpuscles and leptomeninges, difference = 0.192 (−0.014, 0.443).

Chronic inflammation was detected within four of the transitional type lipomas but none of the other groups, although most of these cases had undergone previous lipoma surgery. Despite the larger sample size for transitional lipomas, none demonstrated Pacinian corpuscles. Dermal pits were found in all three subtypes: caudal, dorsal and transitional. Bone marrow was detected only in dorsal lipomas; the significance of this is unclear. There was no further correlation between the different subtypes and degree of cellular diversity and maturity of structures (Figures 4 and 5 and Supporting Information).

## Discussion

Our findings are superficially similar to previous publications with LSLs consisting of mature adipocytes surrounded by thick bands of connective tissue.^9^, ^10^, ^11^, ^12^, ^13^ However, we demonstrate a much higher incidence of a number of key features. Within the thickened bands of connective tissue all cases demonstrated peripheral nerve fibres, and 90.6 ± 7.0% of specimens demonstrated enlarged thickened blood vessels. The uniformity of these additional findings, which are not characteristic of non‐spinal lipomas, raises the question as to whether the term ‘lipoma’ is the most accurate name for this pathology. Supporting previous publications on LSL pathology, we propose the term ‘conus hamartoma’, with LSLs consisting of non‐malignant mature cell types in a disorganised mass with the presence of cell types not usually located in the region.

**Figure 6:**
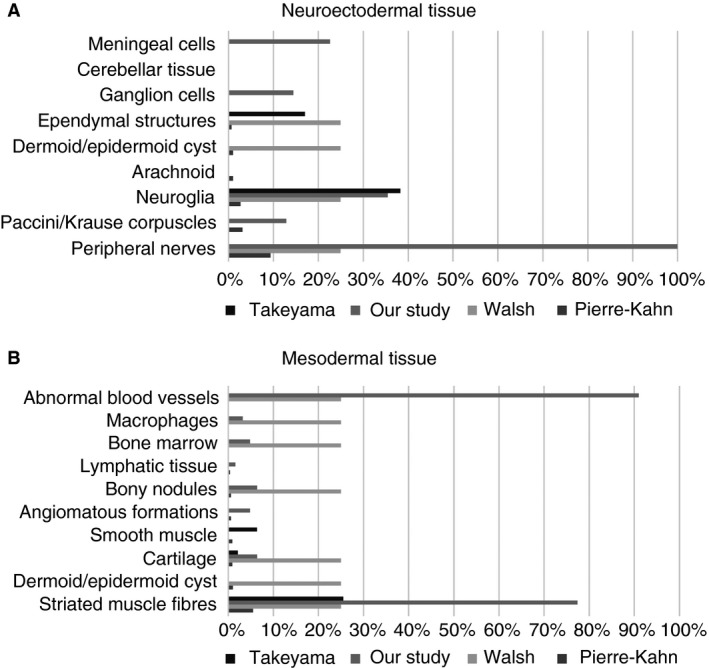
**A**, Comparison with previously published data on frequency of cell types of neuroectodermal origin. **B**, Comparison with previously published data on frequency of cell types of mesodermal origin.

The frequency of particular cell types also differs from other publications (Figure 6). None of our samples included cells of endodermal origin. Our series specifically excluded sacral teratomas, anterior sacral meningocoeles, Currarino syndrome or myelomeningocoele, as these are fundamentally different dysraphic anomalies and would have confounded the results.

Cells of neuroectodermal origin were present with Pacinian corpuscles and ganglion cells occurring with approximately equal frequency, with glial tissue and meningeal cells being identified more frequently than published previously. A large number of cell types identified within LSL specimens may be derived from or associated with neural crest cells. A number of recent publications have demonstrated the ability of neural crest cells to differentiate into an even larger range of cell types than thought previously, including adipocytes and bone marrow.^17^, ^18^, ^19^, ^20^ This raises the possibility that neural crest differentiation may have a role in the embryogenesis of LSL and casts doubt on the previous notion of premature dysjunction.

‘Complex’ LSLs (transitional and chaotic) are related intimately to the conus; indeed, the precise position of the conus may be difficult to identify on MRI in these types. These malformations lie at the interface between primary and secondary neurulation. Some published series indicate that these forms are more likely to result in severe neurological and particularly urological dysfunction.^2^ By contrast, ‘simple’ LSLs (caudal and dorsal) tend to lie on either side, below or above, respectively, the primary–secondary neurulation interface. The conus is identified more readily on MRI in these types of LSL. Although these subtypes seem to present as distinct anatomical entities, there was no correlation with the diversity or maturity of cell types and tissue between complex and simple LSLs (Table 1).

**Table 1:** Comparison of location, radiological and histological features between simple and complex lumbosacral lipomas

	Simple	Complex
Previous classification	Dorsal, caudal^a^	Transitional, chaotic
Characteristic location	Dorsal aspect of conus or caudal aspect of conus	Extending from dorsal to caudal aspect of conus, extending ventrally
Radiological features (MR)	Associated with bony spina bifida	Associated with bony spina bifida
	Preserved conus morphology	Conus poorly delineated
		Rotation of the neural placode
Histological features	Predominantly mature adipocytes	Predominantly mature adipocytes
	Cells of mesodermal and neuroectodermal origin	Cells of mesodermal and neuroectodermal origin

MR, Magnetic resonance.

a Lipomas of the filum terminale with intact conus.

Primary neural tube closure is responsible for formation of the spinal cord above the level of the conus. The remainder of the neural tube, i.e. the conus and filum, forms via secondary neurulation. A mass of neuromesodermal progenitor cells located in the tail bud proliferates to produce a solid rod‐like structure which fuses with the primary neural tube. Subsequent cavitation results in completion of formation of the neural tube.^21^, ^22^, ^23^

As, by definition, caudal and transitional LSLs involve the conus, they are assumed to be related to a defect in the process of secondary neurulation. This is reflected in the high incidence of lipomas associated with syndromes of complex anorectal and urological malformation [OEIS (omphalocele, exstrophy, imperforate anus, spinal syndrome, cloacal extrophy), VATER (vertebrae, anus, trachea, esophagus, renal)], in which maldevelopment of the caudal cell mass and tail bud are implicated.

A number of different hypotheses have arisen over recent decades regarding the origin of LSL, although none of these have been supported by experimental evidence. McLone *et al*. proposed the theory of premature dysjunction whereby the ectoderm and neuroectoderm separate before closure of the neural tube, thus allowing paraxial mesoderm to migrate into the open neural tube preventing closure and differentiating into fat cells.^24^

Catala proposed the hypothesis of incomplete dysjunction whereby the ectoderm never separates completely from the neuroectoderm and forms a dermal tract that subsequently disrupts normal development around the dorsal spinal cord. As a double‐hit model, Catala then proposes that teratogenic cells might be present, inducing abnormal differentiation of the dorsal mesoderm into tissue derived from all three germ layers.^25^

As both these theories involve defects in the process of primary neurulation they can only explain the pathogenesis of dorsal LSLs. In addition, these hypotheses would suggest that a dermal sinus/pit should be associated only with dorsal LSLs.

McLone and Naidich later proposed a role of the tailbud, involved in the process of secondary neurulation, in the formation of caudal LSLs. Similarly, Catala proposed that spinal lipomas associated with sacral agenesis must be due to malfunctions in axis elongation, i.e. the tailbud.^25^ On comparing dorsal LSL (thought to be due to a primary neurulation defect) with the other LSL subtypes (thought to be due to a secondary neurulation defect) we found no significant difference in the type or diversity of cells present, and particularly the presence of a dermal pit. These data therefore challenge these views on embryogenesis of LSL and suggests a unifying mechanism of pathogenesis, rather than the currently proposed models.

The role of secondary neurulation in conus formation, the lack of an established and proven mechanism and the results of this current pathology study highlight the need to re‐examine the theory of embryogenesis of LSL and also our notions of the mechanisms of neurological deterioration. As mentioned above, we propose an alternative embryological origin of spinal lipomas due to maldifferentiation of neural crest cells. Recent literature has indicated that neural crest cells are present within the secondary neural tube as well as the primary neural tube.^26^ In addition, our understanding of the cell biology of neural crest cells is expanding rapidly, with neural crest stem cells being identified in a range of different tissue types.^17^, ^18^, ^19^, ^20^ These cells have the potential to differentiate and even transdifferentiate into the range of cell types seen within the majority of samples within our series.

Historically, all patients underwent early prophylactic surgery due to the pervading assumption that neurological deterioration was inevitable. However, recent evidence suggesting that not all children will ultimately become symptomatic has led to an increasing number of surgeons practising a watch‐and‐wait policy.^2^, ^3^ In keeping with the diverse range of classes and histopathology, clinical deterioration is inconsistent and variable. The presence of mature tissue structures in addition to adipocytes suggests a degree of dysgenesis not described previously, and hints at a cellular mechanism that may contribute to clinical deterioration beyond the mechanical aspect.

The presence of a higher frequency of tissue types within our series may be due to the level of sectioning and analysis. On average, analysis of one block identified 2.9 abnormal cell types, whereas analysis of four blocks revealed on average 4.1 abnormal cell types. Histopathological diagnosis was therefore optimally accurate with analysis of four blocks. With such diverse heterogeneous specimens, it can be assumed that further analysis is likely to reveal further less‐frequent cell types. However, our data suggest that abnormal tissue and cells types are dispersed throughout the lipoma tissue, and sufficient analysis is achieved with reviewing four blocks.

In conclusion, our in‐depth histopathological analysis of LSL highlights the heterogeneity of cell types within all samples that are not reflected in the current nomenclature. We find no histological difference between radiological subtypes refuting previously proposed theories for separate embryogenesis of the different subtypes. The diversity and maturity of cell types also challenges currently held notions, and may have implications for both the mechanisms of clinical deterioration and the role of surgical intervention. Pure lipomas attached to a fundamentally functional spinal cord are more likely to deteriorate through traction and thus may benefit from untethering surgery. By contrast, more diverse, hamartomatous lipomas (particularly those that occur in the context of other caudal cell mass anomalies) with questionable functional integrity of the conus region would be less likely to benefit from surgery.

## Conflicts of interest

None of the authors have any conflicts of interest to declare.

## Supporting information


**Figure S1.** (**A**) Dot plot demonstrating the range of the number of blocks reviewed. (**B**) Mean number of cell/tissue types detected for specimens based on the number of blocks reviewed. Calculated values for 1, 2, 3, 4, 5, 6 and 9 blocks were 3.0, 3.1, 3.6, 4.1, 3.4, 4.0, 0.1.
**Table S1.** Statistical analysis of data Subtypes of lipoma were grouped into those proposed to be due to a defect in primary neurulation (dorsal) and those proposed to be due to a defect in secondary neurulation (caudal, transitional and chaotic).
**Table S2.** Statistical analysis of data Subtypes of lipoma were grouped into ‘simple’ (dorsal and caudal) and ‘complex’ (transitional and chaotic.).
