# Supplementary material for: The pathology of lumbosacral lipomas: macroscopic and microscopic disparity have implications for embryogenesis and mode of clinical deterioration
Source: Histopathology. 2018 Mar 15;72(7):1136–44. doi: 10.1111/his.13469 (PMC5969216; doi:10.1111/his.13469)
Supplement: Supplementary file 1 — Figure S1. (A) Dot plot demonstrating the range of the number of blocks reviewed. (B) Mean number of cell/tissue types detected for specimens based on the number of blocks reviewed. Calculated values for 1, 2, 3, 4, 5, 6 and 9 blocks were 3.0, 3.1, 3.6, 4.1, 3.4, 4.0, 0.1. Table S1. Statistical analysis of data Subtypes of lipoma were grouped into those proposed to be due to a defect in primary neurulation (dorsal) and those proposed to be due to a defect in secondary neurulation (caudal, transitional and chaotic). Table S2. Statistical analysis of data Subtypes of lipoma were grouped into ‘simple’ (dorsal and caudal) and ‘complex’ (transitional and chaotic.). [file HIS-72-1136-s001.docx]

Supplementary data

Figure 7a.


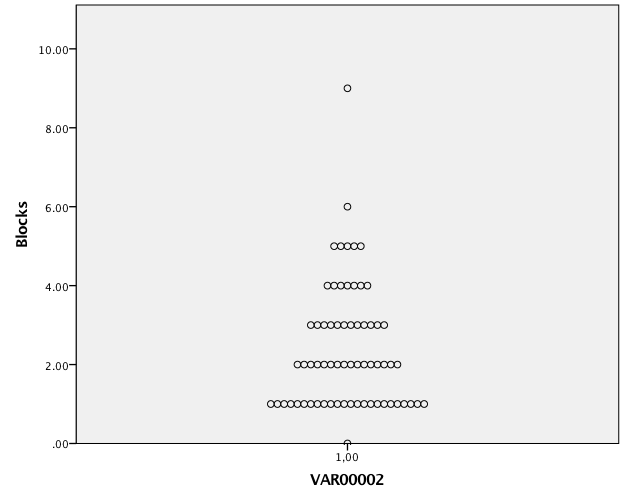


Figure 7b.


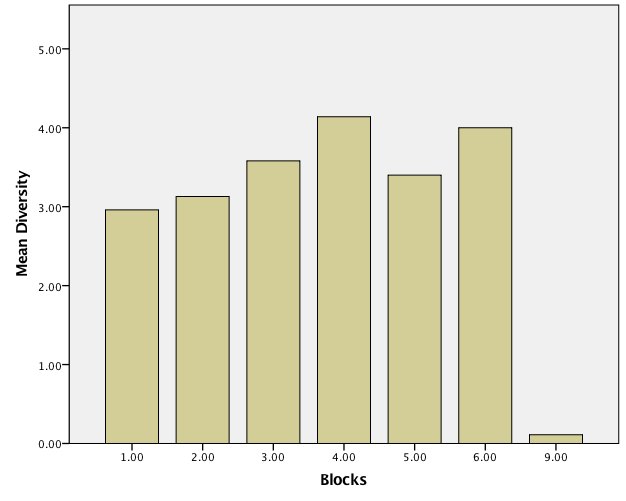


Table 2

| **Subtype** | **Thickened CT** | **Small nerves** | **Large vessels** | **Glial cells in CT** | **Skeletal muscle** | **CNS** | **Ganglion cells** | **Meningeal cell lined cavity** | **Pacinian corpuscule** |
| --- | --- | --- | --- | --- | --- | --- | --- | --- | --- |
| Dorsal | 1.00 | 1.00 | 0.89 | 0.33 | 0.44 | 0.44 | 0.00 | 0.11 | 0.22 |
| Caudal | 1.00 | 1.00 | 0.95 | 0.37 | 0.37 | 0.21 | 0.16 | 0.16 | 0.16 |
| Transitional | 1.00 | 1.00 | 0.89 | 0.33 | 0.59 | 0.07 | 0.07 | 0.07 | 0.00 |
| Chaotic | 1.00 | 1.00 | 0.86 | 0.57 | 0.43 | 0.43 | 0.00 | 0.00 | 0.00 |
| Secondary | 1.00 | 1.00 | 0.91 | 0.38 | 0.49 | 0.17 | 0.09 | 0.09 | 0.06 |
|  |  |  |  |  |  |  |  |  |  |
| Difference | 0.000 | 0.000 | -0.017 | -0.044 | -0.046 | 0.275 | -0.094 | 0.017 | 0.166 |
| 95% CI | -0.299,0.068 | -0.299,0.068 | -0.345,0.125 | -0.296,0.290 | -0.333,0.270 | -0.009,0.574 | -0.203,0.210 | -0.165,0.449 | -0.021,0.493 |

| **Subtype** | **Leptomeninges** | **Ependymal structure** | **Inflammatory cells** | **Abundant peripheral nerve** | **Bone** | **Cartilage** | **Bone marrow** | **Haemangioma** | **Lymph node** |
| --- | --- | --- | --- | --- | --- | --- | --- | --- | --- |
| Dorsal | 0.22 | 0.00 | 0.11 | 0.11 | 0.22 | 0.22 | 0.22 | 0.11 | 0.00 |
| Caudal | 0.21 | 0.11 | 0.11 | 0.11 | 0.00 | 0.00 | 0.00 | 0.00 | 0.00 |
| Transitional | 0.00 | 0.04 | 0.15 | 0.07 | 0.00 | 0.04 | 0.00 | 0.04 | 0.04 |
| Chaotic | 0.00 | 0.00 | 0.00 | 0.29 | 0.14 | 0.00 | 0.00 | 0.00 | 0.00 |
| Secondary | 0.08 | 0.06 | 0.11 | 0.11 | 0.02 | 0.02 | 0.00 | 0.02 | 0.02 |
|  |  |  |  |  |  |  |  |  |  |
| Difference | 0.147 | -0.057 | -0.002 | -0.002 | 0.203** | 0.203** | 0.222** | 0.092 | -0.019 |
| 95% CI | -0.043,0.475 | -0.154,0.245 | -0.147,0.327 | -0.147,0.327 | 0.025,0.529 | 0.025,0.529 | 0.049,0.547 | -0.029,0.417 | -0.099,0.281 |

Table 3

| **Subtype** | **Thickened CT** | **Small nerves** | **Large vessels** | **Glial cells in CT** | **Skeletal muscle** | **CNS** | **Ganglion cells** | **Meningeal cell lined cavity** | **Pacinian corpuscule** |
| --- | --- | --- | --- | --- | --- | --- | --- | --- | --- |
| Dorsal | 1.00 | 1.00 | 0.89 | 0.33 | 0.44 | 0.44 | 0.00 | 0.11 | 0.22 |
| Caudal | 1.00 | 1.00 | 0.94 | 0.35 | 0.35 | 0.18 | 0.18 | 0.12 | 0.18 |
| Simple | 1.00 | 1.00 | 0.92 | 0.35 | 0.38 | 0.27 | 0.12 | 0.12 | 0.19 |
| Transitional | 1.00 | 1.00 | 0.89 | 0.33 | 0.59 | 0.07 | 0.07 | 0.07 | 0.00 |
| Chaotic | 1.00 | 1.00 | 0.86 | 0.57 | 0.43 | 0.43 | 0.00 | 0.00 | 0.00 |
| Complex | 1.00 | 1.00 | 0.88 | 0.38 | 0.56 | 0.15 | 0.06 | 0.06 | 0.00 |
|  |  |  |  |  |  |  |  |  |  |
| Difference | 0.000 | 0.000 | 0.041 | -0.036 | -0.174 | 0.122 | 0.057 | 0.057 | 0.192** |
| 95% CI | -0.129, 0.102 | -0.129,0.102 | -0.138,0.199 | -0.262,0.203 | -0.395,0.077 | -0.081,0.331 | -0.096,0.236 | -0.096,0.236 | 0.045,0.379 |

| **Subtype** | **Leptomeninges** | **Ependymal structure** | **Inflammatory cells** | **Abundant peripheral nerve** | **Bone** | **Cartilage** | **Bone marrow** | **Haemangioma** | **Lymph node** |
| --- | --- | --- | --- | --- | --- | --- | --- | --- | --- |
| Dorsal | 0.22 | 0.00 | 0.11 | 0.11 | 0.22 | 0.22 | 0.22 | 0.11 | 0.00 |
| Caudal | 0.18 | 0.12 | 0.12 | 0.12 | 0.00 | 0.00 | 0.00 | 0.00 | 0.00 |
| Simple | 0.19 | 0.08 | 0.12 | 0.12 | 0.08 | 0.08 | 0.08 | 0.04 | 0.00 |
| Transitional | 0.00 | 0.04 | 0.15 | 0.07 | 0.00 | 0.04 | 0.00 | 0.04 | 0.04 |
| Chaotic | 0.00 | 0.00 | 0.00 | 0.29 | 0.14 | 0.00 | 0.00 | 0.00 | 0.00 |
| Complex | 0.00 | 0.03 | 0.12 | 0.12 | 0.03 | 0.03 | 0.00 | 0.03 | 0.03 |
|  |  |  |  |  |  |  |  |  |  |
| Difference | 0.192 | 0.048 | -0.002 | -0.002 | 0.048 | 0.048 | 0.048 | 0.009 | -0.029 |
| 95% CI | 0.045,0.379 | -0.084,0.214 | -0.169,0.186 | -0.169,0.186 | -0.084,0.214 | -0.084,0.214 | -0.084,0.214 | -0.115,0.161 | -0.149,0.102 |
